# Open Versus Laparoscopic Oncological Resections for Colon Cancer: An Experience at an Average-Volume Center

**DOI:** 10.7759/cureus.70535

**Published:** 2024-09-30

**Authors:** Anca Monica Macovei Oprescu, Bogdan Dumitriu, Mihai Alin Stefan, Constantin Oprescu, Dana Paula Venter, Venter Mircea, Sebastian Valcea

**Affiliations:** 1 Gastroenterology, Emergency Hospital Prof. Dr. Agrippa Ionescu, Bucharest, ROU; 2 General Surgery, Floreasca Hospital, Bucharest, ROU; 3 Pediatric Surgery, Grigore Alexandrescu Emergency Pediatric Hospital, Bucharest, ROU; 4 Surgery, Bucharest Emergency Hospital, Bucharest, ROU

**Keywords:** colon cancer, d2 lymphadenectomy, laparoscopy, oncology, open surgery vs laparoscopy

## Abstract

Although laparoscopy has some limitations related to tumor size or location along the colon, it has been demonstrated that the oncological results are just as good as for open surgery. One can also add to the benefits faster recovery and start of chemotherapy, with lower rates of complications. Our study aimed to compare open surgery to laparoscopy for non-complicated colon tumors operated in an average case-load center and appreciate its feasibility with regards to the T stage, lymph node yield and conversions. The study is retrospective and expanded over four years (January 2020-January 2024). One hundred sixty-two patients were included in the study. We observed that female patients were frequently operated through laparoscopy (p=0.003). T1 and T2 tumors represented the majority of the tumors in the laparoscopy group (p=0.004). No statistical difference existed in terms of lymph node yield. Laparoscopy was avoided for tumors of the splenic angle (p=0.006). Concluding our results, minimally invasive surgery for non-complicated colon cancer is non-inferior to open surgery. Considerable expertise is required to take on more complex cases such as difficult-to-access tumors or large, invasive cancers. It can and should be offered to patients as an alternative to open surgery.

## Introduction

The universal use of minimally invasive surgery (MIS) requires some consideration, especially in oncological diseases of the digestive tract [1]. Lymph node yield, safety margins, adequate exploration of the peritoneal cavity, and trocar site relapse stand out as the main concerns [1]. The general advantages of MIS have been demonstrated and are related to reduced costs and postoperative pain coupled with faster recovery than open surgery [2]. Concerning oncological digestive diseases the Colon Carcinoma Laparoscopic or Open Resection Study (COLOR) represented the first large prospective multi-institutional paper that demonstrated the noninferiority of laparoscopy in colon cancer compared to open surgery and recommended it to be offered to patients as an alternative [3]. Multiple studies since then confirmed that the overall survival (OS) and disease-free survival (DFS) are similar to open surgery including for large T4 tumors or difficult-to-access tumors such as those located at splenic angle [4-6]. These good oncological results together with the advances made in MIS technology such as 4D laparoscopes and new mechanical suture devices have allowed laparoscopy to be widespread and in certain centers become the standard of care. As in open surgery, the dissection and resection of the colon are standardized with respect to the dissection planes that should follow the embryological development of the colon with high vascular ligation and D1, D2, or D3 lymphadenectomy depending on the tumor stage [7,8]. This regulation made it easier for young surgeons to embrace laparoscopy and decrease the learning curve. The quality control of colon resections is done by the histopathology department through the number of lymph nodes extracted. Free axial and circumferential margins in relation to the tumor are also mandatory. Our study aimed to compare open surgery to laparoscopy in an average case-load center and appreciate its feasibility with regards to the T stage of the tumors, lymph node yield and conversions. 

## Materials and methods

One hundred sixty-two patients underwent surgery for colon cancer in the surgical department of Floreasca Emergency Clinical Hospital, Bucharest during a period of four years (01.01.2020-01.01.2024). The study is structured as retrospective, longitudinal, and observational. The patients extracted underwent surgery for uncomplicated colon cancer. Patients with complications such as bleeding, obstruction, metastasis, and peritoneal carcinomatosis were excluded. A total of 26 patients were excluded due to these criteria. The study was approved by the Institutional Review Board (IRB) of the Emergency Clinical Hospital Floreasca (342/22.02.2024) which also oversaw that the ethical standards were respected. All of the patients signed an informed consent for the surgical intervention and anonymous inclusion in possible future research as this was a university hospital. The patients were diagnosed, staged, and investigated according to the European Society of Medical Oncology (ESMO) guidelines. The evaluation included a complete colonoscopy, Computed Tomography (CT) of the thorax, abdomen and pelvis, Magnetic Resonance Imaging (MRI) in the case of rectosigmoid tumors, and biopsy with confirmed histopathological carcinoma. The MultiDisciplnary Board (MTB) approved the therapeutical decisions. The surgical interventions, either open or laparoscopic were carried out with respect to the oncological dissemination principles. 10 cm safety margins proximal and distal from the tumor were obtained and a no-touch principle was applied throughout the surgical intervention. The vascular steps were executed first with high arterial ligation. The surgical interventions were done by four senior surgeons. Eight conversions were recorded. The surgeons completed their learning curve with more than 20 laparoscopic interventions before the study period. The specimens were staged according to the Tumor Node Metastasis (TNM) guidelines. The data was evaluated using the analysis of variance (ANOVA) model. For nonparametric tests, Fischer's test was used. For normally distributed variables t-test was used while correlations were obtained using the Pearson evaluation. p-values under 0.05 were considered statistically significant.

## Results

The median age was similar with 65 (+/- 8.2) in the open arm and 68 (+/-9.3) in the laparoscopy arm (Table 1).

**Table 1:** Patient demographics and pathology results SD: standard deviation, p value marked with bold defines statistical significance

Traits	Laparoscopy	Open	p	
Age	-	-		0.526
Mean	64.6	68.2	-	
Median + SD	65 (+-8.2)	68(+-9.3)	-	
Sex	-	-		<0.003
Male	21	58	-	
Female	30	53	-	
Location	-	-		<0.006
Cecum	8	25	-	
Ascending Colon	4	32	-	
Transverse Colon	4	12	-	
Splenic angle	2	12	-	
Descending Colon	6	17	-	
Sigmoid Colon	20	62	-	
T stage	-	-		<0.004
Tis	0	0	-	
T1	7	5	-	
T2	22	40	-	
T3	24	64	-	
T4a	2	25	-	
T4b	0	13	-	
Retrieved lymph nodes	-	-		-
Mean	15.18	16.82	0.542	
Median	14 +-5.324	15 +-7.472	-	
Invaded lymph nodes	-	-	0.02	
Mean	1.42	3.24	-	
Median + SD	0 (+/-2.542)	1 (+/-5.426)	-	

In terms of gender, the group overall was homogenous but with an overrepresentation of female patients in the laparoscopy arm. Difficult tumor locations (splenic angle) and large invasive tumors were largely avoided in the laparoscopy group such as large T4 tumors with invasion in the surrounding structures like psoas muscle or urinary bladder. These cases were frequently operated through an open approach (p=0.006) with “en block” resections (Table 1). There wasn’t an important difference in terms of absolute numbers between the two arms with regard to the number of lymph nodes harvested. In the open group, there was a mean of 16.82 while in the laparoscopy group, the mean was 15.18 (p=0.542) (Table 1). Lymph node invasion varied according to the T stage. In the open group, there were 3.24 lymph nodes invaded while in the laparoscopy group, there was an average of 1.42. This aspect is explained by the larger tumors operated through the open technique in comparison to the laparoscopy group. The higher the T grade the more likely the lymph nodes were invaded especially in the case of T3 or T4 tumors. Three cases required conversion. There was no statistical significance regarding the axial invasion rate of each of the techniques. In the open arm nine patients were identified with axial invasion and in the laparoscopy arm three patients.

## Discussion

It is clear by now that laparoscopy is non-inferior to open surgery, and this aspect has been demonstrated by multiple high-quality multicenter randomized control studies like the COST and CONTROL trials or more recent population-based trials like the CLASSIC trial [2,9,10]. The usage of MIS for colon cancer is overall increasing worldwide but important disparities exist between countries and centers [11]. MIS is less frequently used in advanced colon tumors such as T3-T4 when compared to T1. This trend was observed no matter the volume of the center [11]. The data from our center confirms this evolution. It also seems that MIS is more often used in female patients than males - an observation also confirmed by our study. This aspect relates most likely to tumor size and the fact that female patients tend to present more promptly to the hospital in the case of symptoms. MIS was avoided in older patients above 75 years mainly due to anesthesia contraindications [12]. Difficult-to-access tumors such as those of the splenic angle are avoided as concerns exist regarding the oncological outcome [13]. After research of the literature, we observed that this location has been constantly excluded from randomised control trials (RCT). Horsey et al. in a retrospective propensity-matched study demonstrated that in selected cases the oncological outcomes for splenic angle tumors can be just as good as open surgery with improved DFS and OS [14]. For advanced T3 and T4 colon tumors, there is controversy related to the option to choose open or MIS. If the tools are available and the surgeon has conquered his/her learning curve, multiple large systematic reviews and propensity-matched score analyses have shown that after MIS the oncological results are similar to open surgery. Fewer postoperative complications exist and there is a shorter time until the start of chemotherapy and return to normal activity [15-17]. In our study, T3-T4 tumors were operated through MIS but the majority of cases in the laparoscopic group were T1 and T2 tumors. The authors acknowledge that surgeon expertise plays an important role when one needs to choose between these therapies. The decision to take on these difficult cases should not be forced by statistics if the surgeon hasn't completed his/her learning curve. In the Floreasca Emergency Hospital, the first laparoscopic intervention was done in 1993 and represented a cholecystectomy. The first laparoscopic colonic resection was done in 2008 (right hemicolectomy). There was a steady increase in the number of surgeons that adopted MIS for digestive surgery. During the last 10 years, there was an increase from 10% to 35% of the cases operated through MIS. The surgical case volume of the specific surgeon will dictate the likeness to operate a patient through MIS. Costs, reintervention rate, and morbidity are lower in the high volume of surgeons than low volume. On our surgical ward, 50% (n=6) of the surgeons operate on colon cancer through laparoscopy and four of them have completed the learning curve with 20 cases. MIS for colon cancer has been slow to be adopted in Romania mainly due to the fact that it was not mandatory in residency and middle-aged surgeons who form the leading structures of surgery did not push for it until more recently. As these skills have now become mandatory in residency, the younger generation will most certainly adopt a steep learning curve. There is debate around the number of cases needed to cover the learning curve, and the numbers vary greatly from 20 to 70 cases [18]. The majority of studies that investigated the learning curve were done in university hospitals with experienced trained surgeons. In small regional hospitals, it was observed that the operative time stabilized after 15 cases [19]. We should mention that the success of an oncological intervention is not to be evaluated only through the operative time. The majority of these studies considered that the learning was complete when the operative time stabilized while the oncological outcomes such as lymph node yield or axial and circumferential margin invasion were rarely taken into consideration. In terms of lymph node yield, an acceptable lymphadenectomy represents proof of a good-quality resection and it is an integral part of the patient's treatment fundamentals that predict OS and DFS [20,21]. Since 2007 a cut-off of 12 lymph nodes is required to accurately stage and further treat a patient with colon cancer [22]. More recent cohort studies have confirmed that this number is also available for metastatic patients. OS and DFS increase according to the number of retrieved invaded lymph nodes [23]. The majority of studies compared the impact of low lymph nodes on overall survival and did not take into account the tumor stage. It seems that the OS and DFS are impacted when under 12 lymph nodes are removed only for T3 and T4 while survival for T1 and T2 tumors is not influenced [24,25]. Older trials like the COLOR study set the cut-off at 10 lymph nodes. In our department, we try to obtain the largest number of lymph nodes irrespective of tumor stage or metastasis by resecting the large feeding vessels as close as possible to the emergence and complete mesocolic excision (CME). The latter seems to offer the best results in terms of lymph node yield and we recommend using this dissection technique when possible. The role of the pathology department and the technique used to count the lymph nodes must also be mentioned. Diverse chemical substances can be used to dissolve the fat from the specimens. These techniques seem to increase the number of lymph nodes identified [26]. In our pathology department, the lymph nodes are dissected and counted individually. The pathologist is the gatekeeper concerning quality control and we strive to maintain good relations with them, make recommendations, and review when necessary the resected segments to ensure continuous growth in the quality of surgical care. We recommend this attitude be adopted by all surgeons.

Limitations

The study is retrospective so the authors could not control how the information was introduced and stored in the IT system. The research is mono-institutional, thus the results can be biased with respect to the respective hospital. The group of surgeons from which the patients were retrieved was not homogeneous in terms of experience since some did not complete the learning curve at the respective time.

## Conclusions

Laparoscopy can and should be offered as an alternative to open surgery for non-complicated colon tumors as the oncological results are non-inferior. Lymph node yield does differ between the two techniques. Whenever possible CME should be practiced as to respect the anatomical planes of dissection. A joint effort should be made with the anatomy department to review the resected specimens if the integrity is in doubt so the necessary correction in technique can be made. T3-T4 tumors or difficult-to-access ones such as those located at the splenic angle can be operated through MIS if the learning curve of the surgeon has been conquered.
